# Resistance of mitochondrial p53 to dominant inhibition

**DOI:** 10.1186/1476-4598-7-54

**Published:** 2008-06-12

**Authors:** Kristina Heyne, Katrin Schmitt, Daniel Mueller, Vivienne Armbruester, Pedro Mestres, Klaus Roemer

**Affiliations:** 1 Internal Medicine I, José-Carreras-Research Center, Bldg. 45.3, University of Saarland Medical School, 66421 Homburg/Saar, Germany; 2 Anatomy and Electron Microscopy, University of Saarland Medical School, 66421 Homburg/Saar, Germany

## Abstract

**Background:**

Mutation of a tumor suppressor allele leaves the second as backup. Not necessarily so with p53. This homo-tetrameric transcription factor can become contaminated with mutant p53 through hetero-tetramerization. In addition, it can be out-competed by the binding to p53 DNA recognition motifs of transactivation-incompetent isoforms (ΔN and ΔTA-isoforms) of the p53/p63/p73 family of proteins. Countermeasures against such dominant-negative or dominant-inhibitory action might include the evolutionary gain of novel, transactivation-independent tumor suppressor functions by the wild-type monomer.

**Results:**

Here we have studied, mostly in human HCT116 colon adenocarcinoma cells with an intact p53 pathway, the effects of dominant-inhibitory p53 mutants and of Δex2/3p73, a tumor-associated ΔTA-competitor of wild-type p53, on the nuclear transactivation-dependent and extra-nuclear transactivation-independent functions of wild-type p53. We report that mutant p53 and Δex2/3p73, expressed from a single gene copy per cell, interfere with the stress-induced expression of p53-responsive genes but leave the extra-nuclear apoptosis by mitochondrial p53 largely unaffected, although both wild-type and mutant p53 associate with the mitochondria. In accord with these observations, we present evidence that in contrast to nuclear p53 the vast majority of mitochondrial p53, be it wild-type or mutant, is consisting of monomeric protein.

**Conclusion:**

The extra-nuclear p53-dependent apoptosis may constitute a fail-safe mechanism against dominant inhibition.

## Background

Lesions that can contribute to cell transformation normally activate the homo-tetrameric transcription factor p53, primarily to stimulate genes whose products cause senescence or apoptosis [1]. In addition, p53 can provoke apoptosis directly through its interaction with key factors of the apoptotic machinery at the mitochondria and in the cytoplasm [2,3]. As a result, ideally the transformation process is ceased. Among the p53 target genes that can suppress cell proliferation, one of the most important is *p21Waf/Cip1 *(*CDKN1A*), whereas *PUMA *(p53 up-regulated modulator of apoptosis) constitutes a prime candidate for a p53-responsive master gene of transcription-dependent apoptosis, at least in some tissues [4,5]. In contrast, transcription-independent apoptosis by p53, which might have evolved to ascertain faithful tumor suppression in the face of lesions that temporarily compromise transactivation [6], involves binding of p53 to Bcl-2 family proteins outside the nucleus. Remarkably, both the transactivation of genes and the interaction with apoptosis regulators are mediated through, and rely upon, the integrity of p53's core DNA binding domain [7,8]. Tumor-derived mutant p53 proteins are thus usually bi-dysfunctional as they are predominantly mutated within this domain.

Stresses such as DNA-damage, oncogene expression, hypoxia, and reactive oxygen can trigger the translocation of approximately 2% of p53 to mitochondria [9] in many primary and some transformed cell types [6,9-12]. Notably, this seems to occur fast and precede transcriptional effects in certain tissues [7,9,10,13,14]. Mitochondrial p53 is primarily present at the outer mitochondrial membrane where it may form, without the help of further factors, a permeabilizing, death-inducing complex [7,12]. Alternatively, it may form complexes with the anti-apoptotic Bcl-2 and BclXL proteins [7,8]. The affinities of these proteins for p53 are higher than for the pro-apoptotic BH123 proteins Bax and Bak; consequently, the latter are freed, form oligomers, and kill the cell. A further pathway may allow pro-apoptotic Bak to be released from the anti-apoptotic Mcl-1 or BclXL proteins upon their association with a distinct site of the p53 DNA binding domain, and then form oligomers and kill the cell [12]. In addition to its mitochondrial action, p53 may bind to cytosolic BclXL and liberate Bax, and may then activate cytosolic monomeric Bax to form lethal oligomers by a mechanisms involving transient interaction of Bax with p53's polyproline-rich domain [15]. Finally, p53 may act through a combined protein interaction and transactivation mechanism: The product of the p53 target gene *PUMA *resolves an inactive cytosolic p53/BclXL complex by binding to a distinct site on BclXL and allows the activation of Bax by free p53 [16]. Which arm of the complex death program is primarily active almost certainly is cell type and context-dependent.

Mutant p53 is present at the mitochondria regardless of apoptotic stimulus [7], suggesting that in contrast to wild-type (wt) p53, a translocation mechanism is active for the mutant proteins regardless of stress, or that the presence of mutant p53 in and by itself constitutes a death stimulus, as is the case with many other oncoproteins. Apart from exhibiting a wt p53-independent oncogenic 'gain-of-function', which at least in part seems to be mediated through inactivating interaction with other pro-apoptotic members of the p53 family (reviewed in [17]), studies of Li-Fraumeni individuals with an inherited mutated allele and of knock-in mice have clearly indicated that mutant proteins can act dominant-inhibitory, either through hetero-oligomerization with wt p53 expressed from the second allele or through the sequestration of limiting factors [18,19]. Clearly, these interactions can compromise the transcriptional activity of wt p53.

Another potent mechanism of dominant inhibition seems to employ target gene promoter occupation by transactivation-incompetent (ΔN and ΔTA-) members of the p53 family. Interestingly, inhibition of transactivation by ΔN-p73 occupying p53 recognition motifs appears to play an important physiological role in the protection of developing sympathetic neurons from p53- and p63-provoked apoptosis (reviewed in [20]), whereas a similar mechanism based on aberrantly spliced *p73 *giving rise to the Δex2/3p73 isoform, can be active in human tumors (for instance, [21]). Here we began to examine to what extent dominant inhibition by the described mechanisms would affect the extra-nuclear apoptotic functions of p53.

## Results

The following studies were performed primarily in human HCT116 colon adenocarcinoma cells. HCT116 is a poorly differentiated, growth factor-insensitive cell line exhibiting microsatellite instability (MIN) caused by deficiency for the essential hMLH1 mismatch repair factor. Many forms of stress except aberrant oncogene expression can stabilize and activate the wt p53 tumor suppressor present in these cells and elicit the expected responses, including apoptosis and cell cycle arrest [22-24]. 5-fluorouracil (5FU), the mainstay chemotherapeutic for colon cancer, induces apoptosis in a p53-dependent manner in HCT116 cells, primarily through the interference of FdUMP with RNA metabolism [23]. α-amanitin, which causes a global transcription inhibition through the initiation of RNA polymerase II degradation, can also provoke HCT116 cell apoptosis. This transactivation-independent cell death was shown to rely on p53 acting at the mitochondria [6].

When exponentially proliferating HCT116 cultures were treated with α-amanitin (10 μM) and analyzed by flow cytometry, the number of cells with a sub-2n DNA content indicative of apoptosis increased with time (see Additional file 1A). However, this increase was not as marked as the one observed by others with the same cell type [6]. In contrast, 5FU (375 μM) induced a robust apoptosis under similar conditions. In agreement with previous reports [6,23,25], both agents provoked apoptosis in dependence of p53 as HCT116 p53-/- cells failed to respond in this manner (see Additional file 1A). Robust cell death also ensued when HCT116 cultures were simultaneously treated with 5FU and α-amanitin. As expected, inclusion of α-amanitin in the drug cocktail blocked the strong stimulation by p53 of the *p21Waf/Cip1 *gene that was normally observed in the presence of 5FU at both the transcript and protein levels (see Additional file 1B). Thus, in accord with earlier findings [6], this shows that 5FU plus α-amanitin can trigger a p53-mediated cell death in HCT116 cultures in the absence of Pol II-mediated transcriptional transactivation. This cell death was apoptotic as it was accompanied by cytochrome c release from the mitochondrial intermembrane space and by caspase 3 activation (see Additional file 1C).

Apoptosis by 5FU, α-amanitin, or both combined (FA hereafter) was preceded by an increased appearance of p53 in the mitochondrial fraction prepared by biochemical cell fractionation (see Additional file 2A). Remarkably and in agreement with previous reports [7,9], HCT116 p53-/- cells retrovirally infected to express p53 conformational mutant 175H or DNA contact mutant 273H, had mutant p53 in their mitochondrial fraction regardless of stress (see Additional file 2B). To confirm the presence of mutant p53 at the mitochondria, immune electron microscopy was performed on the cell lines in the absence of stress, and the number of gold grains at the mitochondria was counted in a blinded study. Additional file 2C shows that the number of mitochondria-associated grains was significantly higher in the cell lines that express p53 when compared to the vector-only cell line (Chi-square test: *P *< 0.001 for 273H and *P *< 0.04 for 175H). As neither mutant can interact with the Bcl-2 family of proteins [7,8] or transactivate genes [18], no enhanced apoptosis was apparent in these cell lines (data not shown).

**Figure 1:**
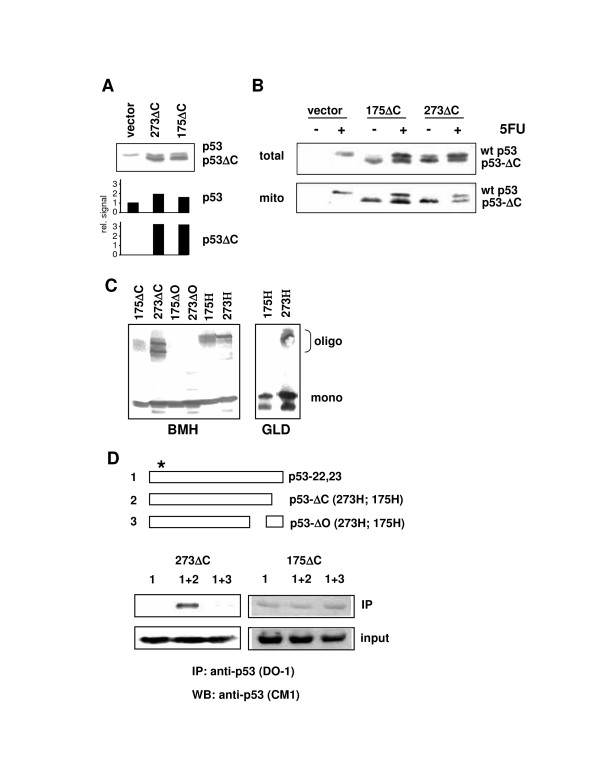
Expression, mitochondrial localization, and oligomerization of mutant p53 proteins. **(A) **HCT116 cells producing endogenous wt p53 were infected with retroviruses to express, from single gene copies per cell, either empty vector or C-terminally truncated 175H and 273H mutants (175ΔC, 273ΔC). p53 was detected with antibody DO-1 (1:2000) applied to 15 μg of total cell extract. The diagram shows the intensities of the signals produced by wt p53 and the truncated proteins, determined by densitometry and with the signal from wt p53 in vector-infected control cells arbitrarily set as one. **(B) **Western immunoblot analysis with anti-p53 antibody DO-1 (1:2000) on total and mitochondrial protein prepared at 24 h after the treatment of the three cell lines with 5FU (375 μM) or not. **(C) **Detection of mutant p53 oligomers in HCT116 p53-/- cells designed to express, again from single gene copies, one of the indicated mutants. The ΔO-mutants carry a deletion in the oligomerization domain. Exponentially growing cells were subjected to crosslinking by either bis-maleimidohexane (BMH) or glutaraldehyde (GLD), as specified in Materials and methods. Oligomers were detected by anti-p53 antibody DO-1 (1:2000) in 15 μg total protein samples that were run on standard SDS-PAGE. **(D) **Hetero-oligomerization between mutant p53 and a full-length p53 with mutated DO-1 epitope (asterisk). HCT116 p53-/- cells were transiently transfected with expression plasmids producing either construct 1 (p53-22,23) plus no protein (empty vector), construct 1 plus construct 2 (mutants 273H or 175H, with truncated C-terminus), or construct 1 plus construct 3 (mutants 273H or 175H, with deleted oligomerization domain). The deletion mutants were immunoprecipitated with antibody DO-1, and could co-immunoprecipitate p53-22,23 only through hetero-oligomerization. p53 was detected in immunoblots with the polyclonal anti-p53 antibody CM-1 (1:500).

To study the effects of mutant p53 on the extra-nuclear, mitochondrial functions of wt p53, parental HCT116 cells (expressing wt p53) were infected, in a first set of experiments and at a multiplicity of infection of <1 colony forming unit per cell, with retroviral vectors expressing either no transgene, or mutants 175H or 273H with a C-terminal 26 amino acid residue truncation (175ΔC and 273ΔC). These truncated proteins were employed to be able to distinguish exogenous and endogenous p53 in Western immunoblots (Figure 1A), and to impair the proteins for potentially confounding gain-of-function activities associated with the C-terminus (e.g. [26,27]). All p53 proteins (endogenous and exogenous) had arginine at amino acid position 72, the major allele among Caucasians and the p53 isoform that is most efficiently translocated to mitochondria [11]. On average the mutant p53 proteins expressed from a single gene copy per cell were produced at 1.5 to 2-fold higher levels than the wild-type in these bulk-infected cultures, and the presence of the mutant protein affected the levels of wt p53 only slightly (Figure 1A). Wt p53 and the truncated mutants were predominantly present in the nuclear fraction (not shown).

Like the full-length mutants, 175ΔC and 273ΔC were present in the mitochondrial fraction of unstressed cells (Figure 1B; compare with Additional file 2B). In contrast, wt p53 translocated to the mitochondria in these mutant p53-transduced cells only after 5FU treatment (Figure 1B). 175ΔC and 273ΔC, like the full-length mutants but unlike mutant proteins with a deletion in the oligomerization domain, were competent for homo-oligomerization, as indicated by chemical crosslinking with *Bis*-maleimidohexane (BMH) that crosslinks sulfhydryl groups, and with glutaraldehyde (GLD) that crosslinks amino groups (Figure 1C). Interestingly, 175H and 175ΔC consistently showed a weaker propensity to homo-oligomerize in this cell type than 273H or 273ΔC, a tendency observed with both BMH and GLD crosslinkings (Figure 1C). This became also apparent in an assay of hetero-oligomerization when the mutants were employed to co-immunoprecipitate, with p53 antibody DO-1, a full-length p53 carrying a mutated DO-1 epitope (Figure 1D). Thus, 273H and 273ΔC seem to form stable oligomers more readily than 175H and 175ΔC in HCT116 cells. Combined, these data indicate that the presence of the mutants 175ΔC and 273ΔC at approximately two-fold increased levels compared to wild-type, does not interfere with the 5FU-induced mitochondrial translocation of wt p53.

**Figure 2:**
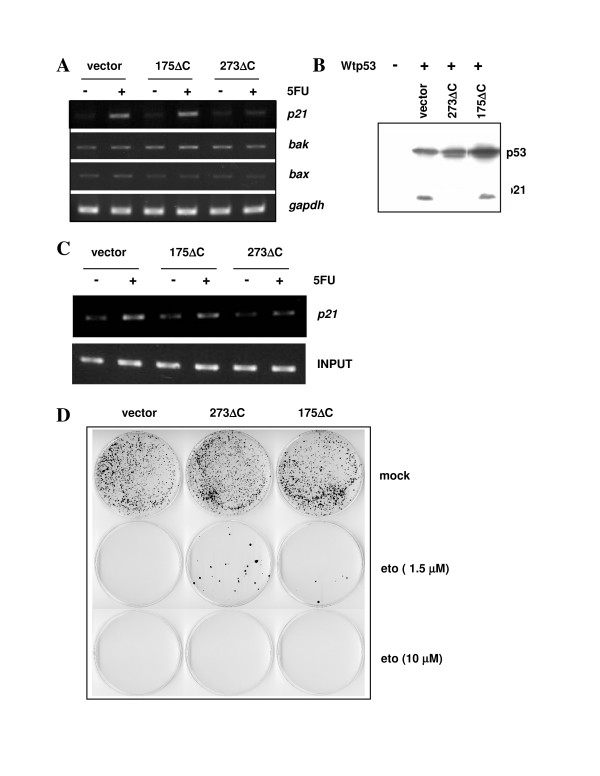
Dominant inhibition of wt p53 by the mutants 175ΔC and 273ΔC. **(A) **RT-PCR on the three HCT116 cell lines infected to express empty vector, 175ΔC, or 273ΔC, after mock-treatment (-) or treatment with 375 mM 5FU (+) for 24 h. The expression levels of the *p21Waf/Cip1*, *bak*, and *bax *genes, and of the *gapdh *control gene, are documented. **(B) **HCT116 p53-/- cells were transiently transfected to express empty vector or wt p53 in combination with one of the indicated mutants. The Western blots on 15 μg of total protein extract prepared at 24 h after transfection were incubated with anti-p53 antibody DO-1 (1:2000) or anti-p21 antibody (1:1000). **(C) **Chromatin immunoprecipitation (ChIP) assay on the three indicated cell lines. Depicted are the relative quantities of *p21 *promoter DNA precipitable with anti-p53 antibody DO-1 at 24 h after treatment with 375 μM 5FU vs. mock treatment. INPUT shows that the total quantities of *p21 *promoter DNA in the cell samples were similar. **(D) **Colony formation assay on HCT116 cells infected with empty vector or vector expressing 175ΔC or 273ΔC. One thousand live cells were seeded onto 10 cm dishes and were mock-treated or received between 10 μM and 0.5 μM etoposide. After 10 days in culture, colonies were fixed and stained with crystal violet, as described in Materials and methods.

We next asked whether the mutants, at the given average levels of expression (see Figure 1A), can act dominant-inhibitory on transactivation upon 5FU treatment. Mutant 273ΔC was able to weaken the 5FU-induced stimulation by wt p53 of the *p21Waf/Cip1 *gene (Figure 2A). *Bax*, *Bak*, and *gapdh *were neither transactivated by p53 nor measurably affected by mutant p53. In accord with its incompetence for oligomerization (see above), mutant 175ΔC failed to act dominant-inhibitory in this assay (Figure 2A), suggesting that hetero-oligomerization rather than competition for limiting factors was the primary mechanism of dominant inhibition by 273ΔC. The discrepancy in dominance between 273ΔC and 175ΔC in HCT116 cells was conserved in transient transfections: 273ΔC inhibited endogenous p21Waf/Cip1 protein expression while 175ΔC did not (Figure 2B).

To confirm the inhibitory effect of 273ΔC on the induced expression of *p21Waf/Cip1*, chromatin immunoprecipitation (ChIP) was performed. Consistent with an inefficient promoter occupation by the hetero-oligomers, the promoter sequence of *p21Waf/Cip1 *was less frequently precipitated with an anti-p53 antibody from cultures expressing 273ΔC than from cultures harbouring vector only. Again, no differences were observed between the 175ΔC and control cells (Figure 2C).

We next asked whether the observed effects of mutant 273ΔC on transactivation might translate into a phenotype. The seeding of 1,000 live cells of the respective cell lines onto 10 cm dishes and the maintenance of these cultures under stress-free conditions resulted in approximately equal numbers of colonies. However, when the cells were exposed to different sub-lethal concentrations of the DNA-damaging drug etoposide, only cells that expressed mutant p53 were able to grow colonies at a threshold concentration of the drug, and again, 175ΔC proved less dominant than 273ΔC in this assay (Figure 2D). Etoposide was chosen as the stressor because it induces primarily cell cycle arrest whereas 5FU induces primarily apoptosis in HCT116 cells [23,28]. Altogether, the data suggest that p53 mutant 273ΔC expressed from a single gene copy and produced, on average, at approximately 1.5-times the level of endogenous wt p53, can act dominant-inhibitory on transactivation and the suppression of colony formation by wt p53 in stressed HCT116 cells. 175ΔC may be impaired in these assays owing to its lower propensity to oligomerize.

**Figure 3:**
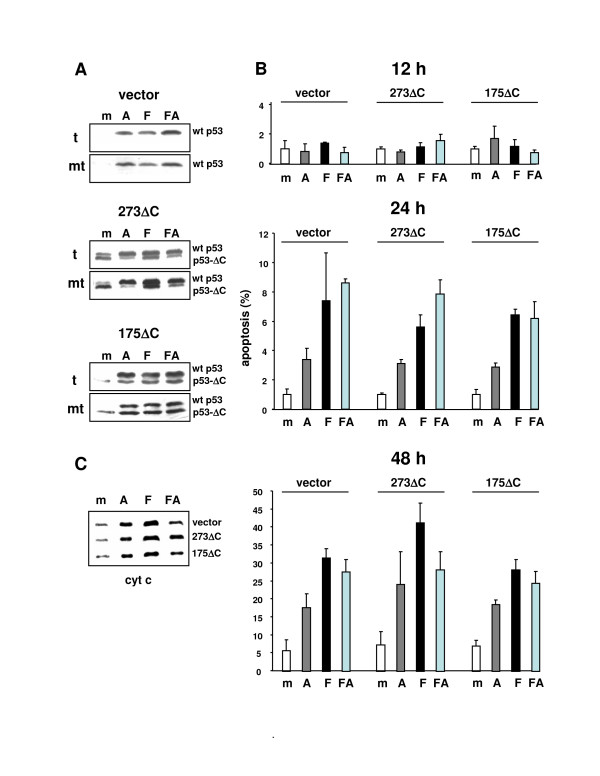
Mitochondrial localization of p53 and apoptosis in HCT116 cells infected to express mutant p53. **(A) **Western blots on 15 μg of total cellular protein (t) or mitochondrial protein (mt) prepared from the indicated cell lines at 24 h after mock-treatment or exposure to α-amanitin (10 μM; A), 5FU (375 μM; F), or both combined (FA). p53 was detected with anti-p53 antibody DO-1 at a dilution of 1:2000. p53-ΔC: one of the indicated ΔC-forms of mutant p53. **(B) **Percentage of apoptosis measured by flow cytometry in cultures of the indicated cell lines. The cells were either mock-treated or drug-treated as specified under. Apoptosis at the 12 and 24 h time points (early apoptosis) was determined by measurement of Annexin V binding as detailed in Materials and methods. The apoptosis at 48 h (late apoptosis) was determined by measurement of the cells with a sub-2n DNA content. **(C) **Western blot on cytoplasm prepared from cultures of the indicated cell lines at 48 h after mock- or drug-treatment. Cytoplasmic accumulation of cytochrome c (cyto c) as a hallmark of apoptosis became apparent after staining of the blot with anti-cytochrome c antibody at a dilution of 1:1000. Vector: HCT116 cells infected with vector-only. 175ΔC: HCT116 cells infected with retroviral vector expressing mutant 175H with a deleted C-terminus. 273ΔC: HCT116 cells infected with retroviral vector expressing mutant 273H with a deleted C-terminus.

Like with the parental HCT116 cells (see Additional file 2A), treatment of the cell lines HCT116-LRNL (vector-only control), HCT116-175ΔC, and HCT116-273ΔC with α-amanitin, 5FU, or both, resulted in the accumulation of endogenous wt p53 in the mitochondrial fraction (Figure 3A). In a next set of experiments, we quantified the apoptosis experienced by the three cell lines by two different means. Cell death provoked by α-amanitin, 5FU, or FA within 12 or 24 h (early apoptosis) was measured flow-cytometrically by the binding of PE-conjugated Annexin V to phosphatidyl-serine on non-fixed cells; necrotic cells were excluded by counterstaining with propidium iodide. Subsequently, DNA fragmentation as an indicator of late apoptosis was quantified by determining the numbers of cells with a sub-2n DNA content in cultures with 48 h of drug exposure. The results are summarized in Figure 3B. Neither drug was able to induce significant apoptosis within 12 h. However, by 24 h and 48 h apoptosis was present in the drug-treated cultures, and a concomitant increase of the cytoplasmic cytochrome c levels was observed in all three cell lines (Figure 3C). Importantly, neither in the cultures that were seeing 5FU alone (p53 at mitochondria, transcription intact) nor in the cultures with α-amanitin (p53 at mitochondria, transcription blocked) was a dominant-inhibitory effect of mutant p53 detectable. This pointed to the possibility that, in contrast to the transactivation of *p21 *(see Figure 2A), the mitochondrial apoptotic function of p53 is resistant to dominant inhibition.

**Figure 4:**
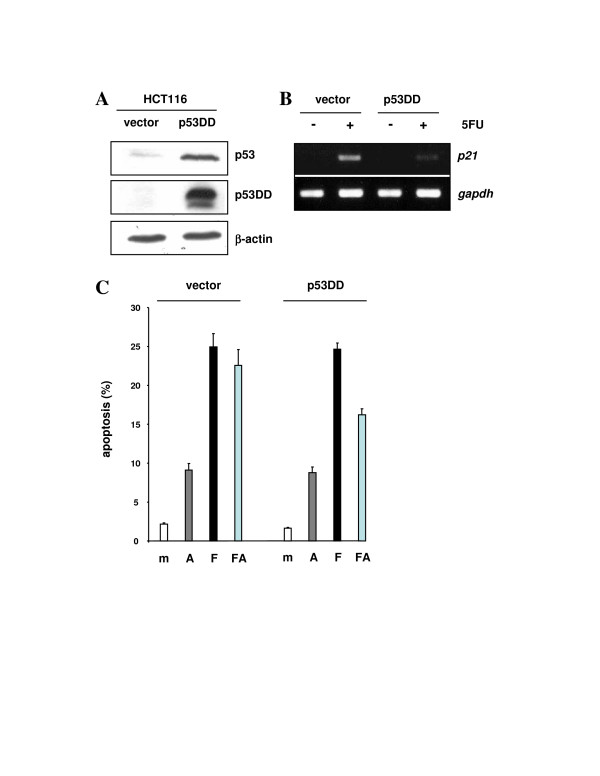
Dominant inhibitory effect of p53DD on transcription but not mitochondrial apoptosis. **(A) **Western immunoblot on 30 μg of total protein extract from HCT116 cells expressing vector-only or p53DD, documenting the expression levels of endogenous wt p53 and p53DD. p53DD was detected with antibody PAb421 (1:200); loading control β-actin was detected with anti-β-actin antibody diluted at 1:5000. **(B) **RT-PCR on total RNA prepared from the indicated cell lines at 24 h after mock-treatment or treatment with 375 μM 5FU. The primers were specific for the p53-regulated *p21 *transcript and for the *gapdh *transcript as a control. **(C) **Percentage of apoptosis in the indicated cell lines and at 48 h after mock-treatment or exposure to α-amanitin (10 μM; A), 5FU (375 μM; F), or both combined (FA). Apoptosis was measured by determining the numbers of cells with a sub-2n DNA content after PI staining, as described in Materials and methods.

To further study whether dominant inhibition might spare the extra-nuclear apoptosis by p53, we resorted to the artificial p53DD mini-protein consisting of the oligomerization domain and a small fragment from the N-terminus (aa 1–14 and 302–390) of mouse p53 [29]. p53DD has been shown to act strongly dominant-inhibitory in cells of mouse and human origin (for instance, [30-32]). We generated HCT116 cells that contain a single copy of the *p53DD *gene by retroviral bulk infection and tested them for dominant inhibition and apoptosis. Figure 4A shows that p53DD is expressed and can induce stabilization of the endogenous wt p53. Figure 4B documents that the stress-induced transactivation of *p21 *is compromised in the HCT116-p53DD cells, in accord with dominant inhibition and with results reported previously [30-32]. Notably, p53DD like 273H failed to inhibit transactivation-independent apoptosis in these cells (Figure 4C), lending further credence to the suggestion that the mitochondrial apoptotic function of p53 is resistant to dominant inhibition in HCT116 cells.

**Figure 5:**
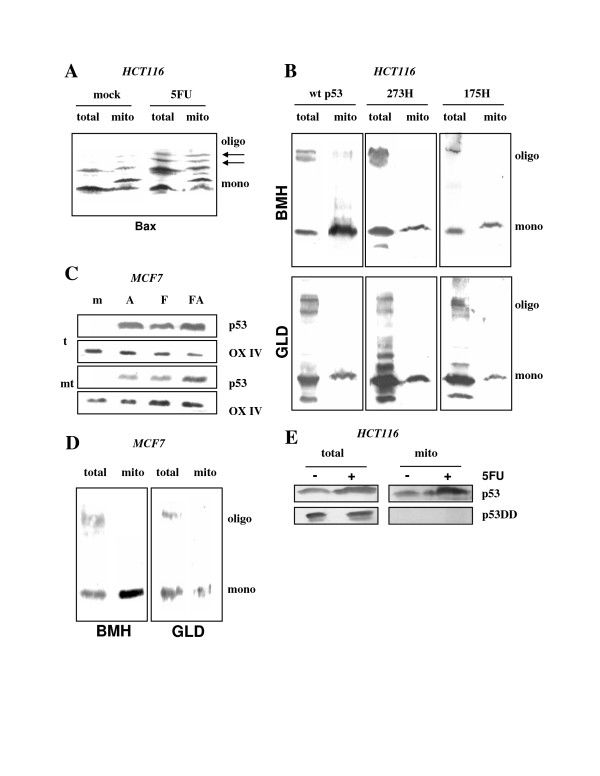
p53 monomers and oligomers in the cell and at the mitochondria. **(A) **HCT116 cells treated with the apoptosis-inducing drug 5FU (375 μM) for 24 h show more oligomers of the apoptotic BH123 protein Bax in the mitochondrial fraction. These were detected in a standard SDS-PAGE after chemical crosslinking with BMH and subsequent immunoblotting with anti-Bax antibody sc-493 at 1:500 dilution (See Materials and methods for details). Total: total cell extract; mito: mitochondrial fraction. Oligo: oligomers; mono: monomers. **(B) **Analogous study of p53 oligomerization in the indicated cell lines and after treatment with 5FU. BMH: bis-maleimidohexane; GLD: glutaraldehyde. Again, oligomers were detected in a standard SDS-PAGE; the anti-p53 antibody was DO-1 (1:2000). wt p53: HCT116 cells infected with vector-only. 273H, 175H: HCT116 p53-/- cells infected with a retroviral vector producing either mutant 273H or 175H. **(C) **Wt p53 in MCF-7 cells translocates to the mitochondria upon treatment with α-amanitin (10 μM; A), 5FU (375 μM; F), or both combined (FA). Cultures were mock-treated or treated as indicated for 24 h, and were then fractionated as described in Materials and methods. 15 μg of total protein (t) or mitochondrial protein (mt) were subjected to Western immunoblot analysis with either anti-p53 antibody DO-1 (1:2000) or anti-cytochrome oxidase IV (OX IV) antibody (1:1000). **(D) **In analogy to (a) and (b), MCF-7 cells were treated with 5FU for 24 h, and p53 oligomerization in the cells and in the mitochondrial fraction was monitored by standard SDS-PAGE and immunoblotting after BMH or GLD crosslinking. p53 was detected with antibody DO-1 (1:2000). **(E) **Mutant p53DD does not translocate to the mitochondria. In HCT116 cells retrovirally infected to express p53DD, endogenous wt p53 is stabilized and mitochondria-associated upon 5FU treatment whereas p53DD is not.

One explanation for the impairment of mutant p53 to interfere with wt p53 at the mitochondria might have been that the mutant cannot efficiently contaminate mitochondrial p53 oligomers through hetero-oligomerization, for instance because the mechanism that translocates wt p53 to mitochondria upon stress is selective for wt tetramers. Notably, we were unable to co-immunoprecipitate mutant and wt p53 from the mitochondrial fraction. We therefore decided to examine the state of p53 oligomerization in this fraction. Consistent with previous findings [33], the presence of Bax oligomers after apoptotic stimulus was readily detectable in total cell protein sample and the mitochondrial fraction by chemical crosslinking with BMH and subsequent immunoblot analysis, documenting that this procedure can detect oligomers in the mitochondrial fraction (Figure 5A). Similarly, p53 oligomers were detected in the total cell protein sample of HCT116 cells expressing either wt p53, 273H, or 175H. In contrast, no or only very few p53 oligomers were found in the mitochondrial fractions of either cell type under identical conditions (Figure 5B). High molecular weight bands indicative of p53 being trapped in megacomplexes were only observed in total cell extracts but not mitochondrial fractions (see Additional file 4), suggesting that a substantial p53 loss through this mechanism is unlikely.

BMH crosslinks sulfhydryl groups while glutaraldehyde (GLD) crosslinks amino groups. To confirm the absence of detectable p53 oligomers in the mitochondrial fraction, a similar study was performed with GLD. As shown in Figure 5B, GLD was able to crosslink p53 in total cell protein samples but not in the mitochondrial fraction. To test whether this was limited to HCT116 cells, we next employed human MCF7 breast adenocarcinoma cells with an intact wt p53 pathway. 5FU induced mitochondrial translocation of p53 in MCF7 cells (Figure 5C). As in HCT116 cells, the mitochondrial fraction of MCF7 cells was devoid of oligomeric p53 upon BMH of GLD crosslinking while oligomers were readily detectable in the total cell protein sample (Figure 5D). Combined, these data suggest that the majority of mitochondrial p53, be it wild-type or mutant, is monomeric. We cannot exclude that mitochondrial p53 consists of unstable oligomers, or that a small fraction of p53 is present at the mitochondria as tetramers and is trapped in megacomplexes after crosslinking. In support of the conclusion that mitochondrial p53 is mostly monomeric, p53DD capable of forming immuno-precipitable hetero-oligomers with wt p53 in HCT116-p53DD cells, was not detectable at the mitochondria whereas wt p53 was (Figure 5E).

For the reasons detailed above, we had employed C-terminally truncated versions of mutant p53 and shown that these, although capable of dominance towards wt p53-mediated transactivation of genes, failed to interfere with the transcription-independent apoptosis by p53 at the mitchondria. To study the effects of full-length mutants, expression plasmids producing HA-tagged full-length 175H, 273H and also 248W as another common tumor-associated conformational mutant were generated. All mutants were expressed to approximately equal levels upon transfection into HCT116 p53-/- cells (see Additional file 3A). In a first study we determined the ratio of mutant vs. wt p53 expression plasmids that knocked down the transactivation by wt p53 of the endogenous *p21 *gene (see Additional file 3B). All mutants including 175H were able to efficiently block p21 activation, indicating that 175H, unlike its truncated variant 175ΔC, was exerting dominance efficiently. This ratio of vector/wt p53 or mutant/wt p53 plasmid was then used to transfect cultures and determine the levels of transcription-independent apoptosis by p53. The results are summarized in Additional file 3C. p53-negative cultures receiving vector-only produced background levels of apoptosis (6–8%). Cultures transfected with mutant p53 plasmids alone showed apoptosis at or even below background (not shown). In contrast, wt p53 expression alone entailed significant cell death (23%). Notably, over-expression of mutant p53 to levels sufficient to inhibit *p21 *transactivation nonetheless allowed significant transcription-independent apoptosis to occur (14–16%). The reduction of apoptosis in cells receiving mutant p53 plasmid in addition to wt p53 plasmid may reflect anti-apoptotic function(s) of over-produced mutant p53. Combined, we interpret these results to support that the transcription-independent mitochondrial apoptosis by p53 in HCT116 cells is relatively resistant to dominant inhibition by mutant p53.

**Figure 6:**
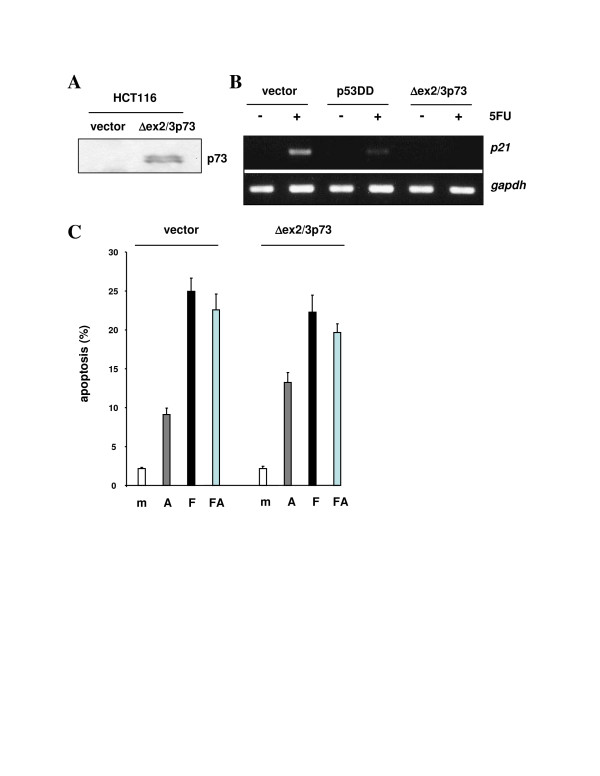
Dominant inhibitory effect of Δex2/3p73 on transcription but not mitochondrial apoptosis. **(A) **Western immunoblot on 30 μg of total protein extract from HCT116 cells expressing vector-only or Δex2/3p73, documenting the expression of Δex2/3p73. p73 was detected with monoclonal antibody cocktail p73Ab-4 (1:200). **(B) **RT-PCR on total RNA prepared from the indicated cell lines at 24 h after mock-treatment or treatment with 375 μM 5FU. The primers were specific for the p53-regulated *p21 *transcript and for the *gapdh *transcript as a control. **(C) **Percentage of apoptosis in the indicated cell lines and at 48 h after mock-treatment or exposure to α-amanitin (10 μM; A), 5FU (375 μM; F), or both combined (FA). Apoptosis was measured by determining the numbers of cells with a sub-2n DNA content after PI staining, as described in Materials and methods.

Dominant inhibition through hetero-tetramerization does not seem to be very efficient, compared with dominant inhibition through target gene promoter occupation by protein isoforms of the p53 family that lack the transactivation domain [34,35]. For instance, the ΔTA-p73 isoforms are among the known strong transcriptional competitors of p53, both in the tumor and normal tissue developmental setting. To test whether tumor-associated Δex2/3p73 [21] can compromise mitochondrial p53-mediated apoptosis, HCT116 cells were retrovirally transduced to express this isoform (Figure 6A). Notably, although the dominant inhibition of the p53-dependent stimulation of *p21 *transcription by Δex2/3p73 was even stronger than the one produced by p53DD (Figure 6B), there was no inhibition of transcription-independent apoptosis (Figure 6C). In summary, our data thus suggest that the extra-nuclear apoptotic functions of p53 at the mitochondria are relatively resistant against dominant inhibition through hetero-tetramerization and promoter occupation.

## Discussion

p53 binds to its DNA recognition motif as a tetramer; monomers can not function as a transcription factor [36]. Multimeric transcription factors, in contrast to monomeric, can be subject to dominant inhibition not only through i) the sequestration of limiting factors by an overproduced mutant, or ii) promoter-occupation by a transactivation-defective isoform, but also through iii) contamination of the multimer by mutated monomers. Although the latter two mechanisms have been documented to be active against p53 *in vitro *and *in vivo *(reviewed in [18,37,38], some major conflicting observations have for some time challenged the concept of dominant-negative action against p53. Doubtlessly, the observation that of the 159 annotated human tumors which had been studied for loss-of-heterozygosity by mid-2007, and which each expressed one out of 90 distinct mutated *p53 *alleles, approximately 60% have lost the wild-type allele (Olivier and Hainaut in [39]), makes a case against the global importance of dominance. Similarly negative was the observation that approximately 50% of the tumors that develop in Li-Fraumeni patients with one inherited mutated full-length p53, have lost their wild-type allele [40,41]. On the other hand, there are several important observations in strong favour of dominance. For example, while only 50% of the Li-Fraumeni tumors with a mutated full-length p53 are wt p53-deficient, this is true for almost 100% of the Li-Fraumeni tumors with an inherited *p53 *allele mutated to produce no protein [40,41].

Strong support for dominant inhibition comes also from studies on transgenic mice, although in this system dominance can be somewhat more difficult to assess, mostly for two reasons. First, the two p53 mutations introduced into the mouse germ line by knock-in in two central studies [42,43], 172H and 270H (corresponding to human 175H and 273H, respectively), display additional, wt p53-independent oncogenic functions (gain-of-function) that may superimpose dominant-negative effects. Second, whereas human p53+/- cells almost always loose their wt allele in the course of transformation, p53+/- cells from mice often retain it [44] indicating that in mice, in contrast to humans, gene dose reduction can impair critical p53 functions to a degree as to mimic, with respect to tumor development, complete p53-deficiency. Nonetheless, careful studies of the efficacy of known wt p53 activities in primary fibroblasts from 172H/+ and 270H/+ mice, compared to cells from -/+ animals, have clearly demonstrated that the mutant proteins can act dominant-negative [42,43]. Combined, the available data clearly indicate that hetero-tetramerization can incapacitate wt p53 as a transcription factor [1,18,37,38]. The degree of inhibition by a mutant depends on the mutant-to-wt protein levels, the site of mutation, and the cell type [34,35,39].

Here we have presented evidence suggesting that the extra-nuclear, mitochondrial death pathway of p53 protein can be resistant to dominant inhibition because mitochondrial p53 is mostly monomeric. This raises important questions. For example, can monomeric p53 trigger mitochondrial apoptosis? Moll and colleagues have convincingly demonstrated that a mitochondrially targeted monomeric p53 was not only capable of provoking apoptosis but was indeed the most efficient cell death inducer of all tested p53 proteins [9]. That group also showed that mitochondrially targeted p53 can induce apoptosis in cells expressing mutant p53 [45]. So clearly, monomeric p53 at the mitochondria can trigger apoptosis.

If the p53-mediated mitochondrial apoptosis is resistant to dominant inhibition, why are the 40% of tumors with a mutant p53 allele that have retained the wt allele (dominant inhibition active) not dying through this pathway? Several aspects may be relevant here. First, such tumors may have evolved to impair this pathway through other means, as for instance the overproduction of Bcl2/BclXL or down-regulation of Bax/Bak. Second, even in cells expressing mutant p53, the translocation of wt p53 to the mitochondria required a stress signal (see Figure 1B). Therefore, (some) p53-dependent apoptosis may well occur in these tumors despite of the inhibition of p53's nuclear transactivator function by the mutant, but this may need stress, as for example under chemotherapy, to become apparent. The HCT116 cells used in this study had to be exposed to genotoxic stress for p53 translocation to occur; the oncogenic deregulation present in these cells was insufficient to act as p53 stressor, probably primarily because HCT116 cells fail to express the oncogenic stress signal mediator p14ARF [46]. Third, Chipuk and colleagues have documented that cell types exist in which efficient mitochondrial apoptosis by p53 is dependent upon the transactivation of the p53 target gene *PUMA*. PUMA resolves an inactive cytosolic p53/BclXL complex and allows the activation of pro-apoptotic Bax by free cytoplasmic p53 [16]. Thus, if tumors that co-express wt and dominant-negative mutant p53 belong to this cell type, extra-nuclear apoptosis induction by p53 might be impaired despite of the resistance of mitochondrial p53 to dominant inhibition by hetero-tetramerization. Forth, some of these PUMA-sensitive tumors may have developed from cell types that are able to mobilize the p53-relatives p63 or p73 for the transactivation of *PUMA*. In such tumors, it will make a difference whether the expressed mutant p53 is of class I (wild-type conformation) or class II (conformation-altered) type. Class II but not class I mutants can bind and inhibit p63 and p73, which is considered to be one mechanism of 'gain-of-function' [17]. Consequently, tumors of this type with the genotype (wt p53/class II mutant), but not (wt p53/class I mutant) tumors, may exhibit a gain-of-function phenotype with respect to the impairment of mitochondrial apoptosis. Importantly, these inferences can be subject to experimental testing. In sum, the resistance of mitochondrial monomeric p53 to dominant inhibition by hetero-tetramerization should increase the selection pressure on a stressed tumor with a mutated *p53 *allele to loose the mitochondrial apoptosis function.

Contamination of the wt p53 tetramer with mutant p53 through hetero-tetramerization is a remarkably inefficient way to inactivate p53 [34,35] whereas dominant inhibition through transactivation-defective (ΔN or ΔTA) isoforms of, for instance, p53 or p73, is very efficient ([21,35], see also Figure 6). The latter mechanism can involve the formation of hetero-tetramers between wt p53 and ΔN-p53, where one truncated protein per tetramer is sufficient to incapacitate it for transactivation [35], and can involve competitive promoter-occupation by ΔN-p53 or ΔN-p73 [21,35]. The extraordinary efficiency of this inhibitory mechanism may be one reason for the fact that it has gained important roles in several physiological processes. For example, ΔN-p73 is a very efficient and perhaps the primary inhibitor of p63-mediated apoptosis during neuronal development, and of p53-mediated apoptosis in response to adult neuron injury and neurodegeneration [20]. With this efficacy of inhibition, it is no surprise that ΔTA-isoforms of p73 have also been found in tumor cells. One might thus speculate that the ΔTA-p73-resistant apoptotic function of mitochondrial p53 has evolved, at least in some cell types, as part of a fail-safe mechanism against the powerful dominant-negative effects of these molecules.

Monomeric p53 not only escapes dominant inhibition through hetero-tetramerization but also ubiquitylation through the E3/E4 ubiquitin ligase MDM2, as MDM2 binds exclusively to tetrameric p53 [47]. On the other hand, (multi-)mono-ubiquitylation of p53 by MDM2, as opposed to poly-ubiquitylation associated with p53 degradation, can promote mitochondrial p53 translocation [48], and in accord with this, the apoptosis- and tetramerization-defective p53 mutant 337C [49] occurring in Li-Fraumeni patients fails to translocate to the mitochondria upon stress (our unpublished observation). Thus, the recent finding that cytoplasmic p53 – mono-ubiquitylated by cytoplasmic MDM2 – travels to the mitochondria without shuttling MDM2 [48], might suggest that monomerization of p53 occurs in stressed cells somewhere between this modification and p53's final destination at the mitochondria.

## Conclusion

Mutant p53 can compromise transactivation by wild-type p53 through hetero-tetramerization, and p53/p63/p73 isoforms lacking a transactivation domain through competitive promoter-occupation as well as hetero-tetramerization. p53 has evolved to command a second, transcription-independent pathway of apoptosis induction at the mitochondria. This second pathway – although probably less efficient than full apoptosis by p53, provoked by both pathways in concert, – is resistant against dominant inhibition in HCT116 cells. Moreover, mitochondrial p53 – in contrast to nuclear – is mostly monomeric. Thus, the extra-nuclear p53-dependent apoptosis may constitute a fail-safe mechanism against dominant inhibition.

## Methods

### Plasmids, chemicals, and antibodies

pLRNL-175ΔC, pLRNL-175ΔO, pLRNL-273ΔC, pLRNL-273ΔO pLRNL-175H and pLRNL-273H have been described elsewhere [50]. Full-length mutant p53 genes 175H, 273H and 248W were cloned into expression plasmid pCMV-pA. Plasmid pLXSNp53DD was kindly provided by Moshe Oren, Rehovot, Israel. Plasmid p73Δ2/3 β-pcDNA-3.1 was kindly provided by Thorsten Stiewe, Würzburg, Germany. The p73Δ2/3 β insert was cut out with BamHI and subcloned into pLRNL. The drugs α-amanitin, 5-fluorouracil (5-FU) and etoposide were from Sigma (St Louis, USA), as were propidium iodide for DNA content analysis, glutaraldehyde and crystal violet for fixation and colony staining, the β-actin monoclonal antibody, and the peroxidase-conjugated secondary anti-mouse and anti-rabbit antibodies. The crosslinker BMH was from Pierce (Rockford, USA). G418 and the transfection reagent Nanofectin I were purchased from PAA (Pasching, Austria). The p53 monoclonal antibodies DO-1 and PAb421, and puromycin were purchased from Calbiochem (San Diego, USA); the p53 polyclonal antibody CM-1 was from Biocare Medical (Pike Lane, USA); the p73Ab-4 antibody cocktail (clones ER-13 + ER-15 +GC-15) was purchased from Lab Vision Corporation (Fremont, USA); the monoclonal cytochrome c antibody (clone 7H8.2C12) was from BD Biosciences (Franklin Lakes, USA); the cytochrome c oxidase subunit IV monoclonal antibody (10G8) was from Molecular Probes (Eugene, USA) and the anti-HA antibody 12CA5 was from Sigma (St. Louis, USA). The monoclonal caspase-3 antibody (8G10) was purchased from Cell Signaling Technology (Boston, USA). The monoclonal p21 antibody was from BD Pharmingen (Franklin Lakes, USA). The polyclonal Bax antibody (sc-493) was acquired from Santa Cruz Biotechnology (Santa Cruz, CA). Protein G Sepharose (4 Fast Flow) was from GE Healthcare (Uppsala, Sweden). The Annexin V:PE apoptosis detection kit was purchased from BD Biosciences (Franklin Lakes, USA).

### Cell culture, viruses, and transfection

293GP retrovirus producer cells, MCF-7, and H1299 cells were cultured in DMEM, and HCT116 cells and derivatives in McCoy's 5A medium, all supplemented with 10% FCS and grown in a humidified 7% CO_2 _atmosphere at 37°C. Retroviruses were produced in the 293GP producer line after a 1:1 transfection with a vector coding for the stomatitis virus G protein and either empty retroviral vector or vector expressing one of the p53 mutants, p53DD, or Δex2/3p73. The viruses were harvested 48 h after transfection. The supernatant was filtered through a 0.45 μM filter (Renner, Germany). Virus stocks were titered on 293 cells and frozen in aliquots at -80°C. For infection, cultures were incubated with virus stocks at a multiplicity of infection (MOI) of approximately 0.1 colony-forming units (cfu)/cell to assure transfer of only one transgene per infected cell. Infection was carried out in the presence of 4 μg/ml polybrene (Sigma) for 4 h, and virus-infected cells were selected in 400 μg/ml G418, beginning at 48 h after infection, for approximately 7 days. Exponentially growing cultures that had been out of selection for at least 2 days were drug-treated as outlined in the main text and figure legends. As transfection reagent, Nanofectin I from PAA (Pasching, Austria) was employed according to the manufacturer's recommendation.

### Flow cytometry analysis of DNA content and apoptosis

Twenty-four h prior to drug treatment, cells were seeded in six-well dishes to approximately 30% confluency. At the indicated time points, the cells on the dishes were harvested by trypsinization, washed in PBS, resuspended in 200 μl of 0.9% NaCl, squeezed through a 23.5-gauge needle into 1.8 ml of methanol, and fixed overnight at -20°C. Cells were resuspended in PBS supplemented with RNase A (25 μg/ml) at approximately 10^6 ^cells per ml, and were stained with PI (25 μg/ml) for >1 h at 4°C. DNA fluorescence was measured with a Becton Dickinson FACSCanto (Bedford, USA) and the data were analyzed with BD FACSDiva software from Becton. FACS detection of Annexin V:PE at apoptotic cells was done according to the protocol supplied with the Annexin V:PE apoptosis detection kit from BD Biosciences (Franklin Lakes, USA).

### Colony formation assay

For colony formation assays, 10^3 ^live cells (counted with a FACSCanto) were seeded onto 10-cm dishes and grown for 24 h. The cultures were then incubated in the presence of etoposide for 10 days. Colonies were washed with PBS, fixed with 1.25% glutaraldehyde for 20 min, washed again and stained with 1% crystal violet in PBS for 1 h at room temperature.

### Preparation of subcellular fractions and immunoblot analysis

Mitochondria protein fractions were prepared and tested essentially as described before [25]. In brief, mitochondria were isolated with the Mitochondria Isolation Kit for Mammalian Cells (Pierce, Rockford, USA), following the supplier's protocol. The centrifugation step at 700 g was repeated three times. Mitochondria were lysed in an SDS-lysis buffer heated to 100°C, containing 100 mM Tris-HCl (pH 6.8), 100 mM DTT, 4% SDS, and 20% glycerol. 15 μg of mitochondrial protein were subjected to 8 or 13% SDS-PAGE and analyzed by Western blotting. For immunoblot analysis, cells were lysed in the SDS-lysis buffer heated to 100°C. Samples containing 15 or 30 μg of total cellular protein were subjected to 8, 10, or 13% SDS-PAGE and transferred to a PVDF membrane (Immobilon-P; Millipore, Bedford, USA). Signals were detected upon overnight incubation of the membranes with one of the indicated antibodies, followed by a final incubation with a peroxidase-conjugated secondary anti-mouse (1:2000) or anti-rabbit (1:2000) antibody and Pierce ECL Western Blotting Substrate (Rockford, USA), performed as specified by the supplier.

### Cytochrome c release

Cells were treated with the indicated drugs, and nuclei and mitochondria were again separated from the cytosolic fraction with the Mitochdria Isolation Kit for Mammalian Cells from Pierce (three centrifugation steps at 700 g, one centrifugation step at 3000 g, one centrifugation step at 12000 g). The cytosolic fraction was then concentrated using Microcon^® ^Centrifugal Filter Devices YM-10 (Millipore, USA) for 10,000 nominal molecular weight limit, performed as specified by the supplier. Concentrated samples were mixed with SDS-lysis buffer, heated to 100°C, and analyzed by Western blotting.

### RNA analysis

Cells were seeded in 10 cm-dishes and treated 24 h later with 5-FU. One day after drug-treatment medium was removed and solution D (236.4 g guanidinium thiocyanate; Sigma, USA, in 293 ml water, 17.6 ml 0.75 M sodium citrate pH 7.0, and 26.4 ml 10% sarcosyl, 0.72% 2-mercaptoethanol) was added. Cells were scraped off and 0.1 ml of 2 M sodium-acetate pH 4, 1 ml of water-saturated phenol (Roth, Germany), and 0.2 ml of chloroform-isoamylalcohol (49:1) were added, mixed, and cooled on ice for 15 min. After centrifugation (10,000 g, 4°C, 20 min) the aqueous phase was collected and precipitated with isopropanol overnight. After a further centrifugation (10,000 g, 20 min, 4°C), RNA was redissolved in solution D and precipitated with isopropanol at -20°C for 1 h. The pellet was washed in 70% ethanol and dissolved in DEPC-water. The RNA was digested with RNase-free DNase I (Roche, Germany) for 60 min at 37°C, and 4 μg was used for the first-strand cDNA synthesis with SuperScript™III (Invitrogen, USA) as specified by the manufacturer. Semiquantitative RT-PCR analysis was performed with HotStarTaq (Qiagen, Germany), using the primers: *p21 *(for: ggcggcagaccagcatgacagatt; rev: atgaagccggcccacccaacctc; T_A_: 64°C), *bak *(for: taggcgctggggagactgataact; rev: aggcttggaggcttctgacacg; T_A_: 65°C), *bax *(for: ccccgagaggtctttttccgagtg; rev: gaaaaatgcccatgtcccccaatc; T_A_: 65°C), and *gapdh *(for: tggtatcgtggaaggactcatgac; rev: agtccagtgagcttcccgttcagc; T_A_: 64°C).

### Immune electron microscopy

Whole cells were fixed in 4% formaldehyde/0.05% glutaraldehyde, dissolved in 0.1 M cacodylat buffer (pH 7.4) at RT, and stored overnight at 4°C. Pellets were resuspended in 2% low-melting point agarose at 40°C and solidified on ice. Whole cells were fixed to the agarose gel with the formaldehyde/glutaraldehyde fixative (see above). After washing the gel with 0.1 M phosphate buffer pH 7.2, small blocks (maximum 2 × 2 × 2 mm^3^) were cut out and dehydrated by the processive-lowering-of-temperature-method, using the following ethanol series and temperatures: 30%, 0°C; 50%, -20°C; 70, 90, 100% at -35°C; for 1 h each. Dehydrated gel blocks were infiltrated and embedded with the acrylate resin Lowicryl K4M (Polysciences, Eppelheim, Germany) at -35°C. The resin was UV-polymerized for 1 day at -35°C, 1 day at 0°C, and 1 day at RT. Ultrathin sections (70–80 nm) were placed on droplets (30 μl) of the following: glycine (50 mM in PBS); blocking solution; anti-p53-antibody DO-1 or IgG control diluted in blocking solution; blocking solution; goat anti-mouse antibody coupled to 10 nM colloidal gold (Aurion, Netherlands); blocking solution; PBS; 2.5% glutaraldehyde in 0.1 M phosphate buffer; PBS; and water. The blocking solution contained 0.5% cold water fish gelatine, 0.5% BSA, 0.01% Tween-20 (all from Sigma), dissolved in PBS. The incubations with the antibodies were carried out overnight at 4°C in a wet chamber. Finally, the sections were dried and stained with uranyl acetate and methylcellulose. All intact mitochondria detected at 68,000× magnification in randomly chosen fields were analysed with morphometric software (Analysis, SIS, Germany).

### Chromatin immunoprecipitation and protein co-immunoprecipitation

Cells were seeded and 24 h later treated with 5-FU. The ChIP analyses were performed with the Chromatin Immunoprecipitation Assay Kit from Upstate (Lake Placid, USA) according to the manufacturer's recommendation, with the following modifications. 2.5 μg p53-antibody (DO-1) or irrelevant antibody, linked to Protein G sepharose 4 Fast Flow™, were used for immunoprecipitation. For PCR, 2 μl out of 50 μl of DNA extractions were employed. The primer sequences and PCR conditions used to amplify the corresponding promoter fragments were as follows: *p21 *(for: acctttcaccattcccctac; rev: gcccaaggacaaaatagcca; T_A_: 56°C); *U6 *(for: ggcctatttcccatgattcc; rev: atttgcgtgtcatccttgc; T_A_: 56°C). For protein co-immunoprecipitation, HCT116 cultures at a density of approx. 50% were transiently transfected by Nanofectin I (PAA, Austria) with the different expression plasmids, and 24 h later the truncated p53 protein with the intact DO-1 epitope was immunoprecipitated with antibody DO-1, following our standard IP protocol [51]. The full-length p53 with a defective DO-1 epitope that was co-precipitated along with the truncated p53 could be detected in a Western blot with the polyclonal p53 antibody CM-1.

### Chemical crosslinking

Total protein extracts were prepared by lysing the cells in a buffer containing 10 mM Tris pH 7.6, 140 mM NaCl, 0.5% Nonidet P-40 (NP-40), and proteinase inhibitor cocktail (Sigma, USA). After incubation for 30 min at 4°C the samples were centrifuged at 13.000 rpm for another 30 min at 4°C. For BMH crosslinking, whole cell lysate was incubated with 0.2 mM BMH (bis-maleimidohexane; Pierce) according to the provided protocol. After an incubation of 1 h at RT, monomers and multimers were separated on SDS-polyacrylamide gels and detected with p53 antibody DO-1 or Bax antibody sc-493. Mitochondria prepared with the Mitochondria Isolation Kit for Mammalian Cells (Pierce), were resuspended in the BMH conjugation-buffer and incubated with BMH (final concentration: 1 mM) at 37°C for 1 h. For crosslinking with glutaraldehyde (GLD), whole cell lysates were incubated with 0.0025% GLD for 15 min at RT, as described before [49]. Isolated mitochondria were resuspended in PBS and incubated with 0.0025% GLD for 15 min at 37°C. Finally, monomers and multimers were again separated on SDS-polyacrylamide gels and detected with the respective antibodies.

## Competing interests

The authors declare that they have no competing interests.

## Authors' contributions

KH, KS, and DM generation of cell lines and analysis of mitochondrial apoptosis, VA ChIP analyses, PM immune electron microscopy, KR study design and supervision, manuscript preparation. All authors read and approved the final manuscript.

## Supplementary Material

Additional file 1 *α-amanitin and α-amanitin plus 5-fluorouracil can provoke a p53-mediated, transactivation-independent apoptosis in HCT116 cells*. **(A) **Exponentially growing cultures of HCT116 and HCT116 p53-/- cells were mock-treated (m) or exposed to α-amanitin (10 μM; A), 5FU (375 μM; F), or both combined (FA). At the indicated time points, the percentage of apoptotic cells was determined by measuring the numbers of cells with a sub-2n DNA content after PI staining, as detailed in Materials and methods. **(B) **RT-PCR were performed on RNA from HCT116 cells after 24 h of drug treatment, as indicated, and the relative levels of the p53-responsive *p21 *and the control *gapdh *transcripts were determined. Western blot analysis confirmed the lack of stimulation of p21 under conditions of transactivation repression by α-amanitin. p53 was detected with DO-1 at a dilution of 1:2000; loading control β-actin was detected with anti-β-actin antibody diluted at 1:5000, and p21 was detected with anti-p21 antibody diluted at 1:1000. **(C) **Induction of apoptosis in HCT116 cells by 5FU and α-amanitin plus 5FU is mediated by cytochrome c (cyt.c) release and caspase 3 (casp3) activation. 15 μg of cytoplasmic protein (for cyt.c) or 30 μg of total protein (for casp3) from cells treated in the indicated way for 48 h were subjected to immunoblot analysis. Anti-cytochrome c antibody and anti-caspase 3 antibody (detecting both the pro-caspase and the activated caspase) were diluted at 1:1000. Click here for file

Additional file 2 *Presence of wt and mutant p53 in the mitochondrial fractions of HCT116 cultures in dependence of treatment*. **(A) **HCT116 cultures were mock-treated (m) or treated with 10 μM α-amanitin (A), 375 μM 5FU (F), or α-amanitin plus 5FU (FA) for 24 h; the cells were fractionated and the quality of the fractionation was tested as described in Materials and methods. 15 μg of total protein (t) or mitochondrial protein (mt) were subjected to Western immunoblot analysis with either anti-p53 antibody DO-1 (1:2000) or anti-cytochrome oxidase IV (OX IV) antibody (1:1000). **(B) **HCT116 p53-/- cells were bulk-infected with retroviruses at a multiplicity of infection of <1 pfu/cell to express either p53 full-length mutant 175H or 273H from single gene copies per cell. The cells were mock-treated (-) or treated with 375 μM 5FU for 24 h (+), and where then fractionated and analyzed by immunoblotting (15 μg protein). total = total cell extract; cyto = cytoplasmic; mito = mitochondrial fraction. Again, proteins were detected with anti-p53 and anti-cytochrome oxidase antibodies. **(C) **Immune electron microscopy detects mutant p53 at the mitochondria of HCT116 p53-/- cells retrovirally infected to express empty vector, 175H or 273H. Gold grains (10 nm; arrows) indicative of the binding of anti-p53 antibody DO-1 were more frequently detected at mitochondria (mt) in cells harboring mutant p53 than in cells with no p53. The diagram outlines the numbers of grains counted in a blinded study at 105 mitochondria from randomly chosen microscopic fields; *P *values were determined with the Chi-square test. Click here for file

Additional file 3 *Effect of full-length mutant p53 over-production on wt p53-mediated p21 expression and transcription-independent apoptosis*. **(A) **HCT116 p53-/- cultures were transfected with empty vector or vector expressing HA-tagged full-length versions of 175H, 273H and 248W. p53 was detected with monoclonal anti-HA antibody 12CA5 (1:1000); loading control β-actin was detected with anti-β-actin antibody diluted at 1:5000. **(B) **Transient transfection of HCT116 p53-/- cultures with vector alone, or wt p53 plasmid plus vector plasmid or mutant p53 plasmid at a ratio of 1:3. p53 was detected with antibody DO-1 (1:2000); mutant p53 was detected with anti-HA antibody 12CA5 (1:2000). Loading control β-actin was detected with anti-β-actin antibody diluted at 1:5000. **(C) **Exponentially growing HCT116 p53-/- cultures were transfected as in B, but under inclusion of 0.1 μg plasmid expressing green fluorescent protein (pC-EGFP) to allow an estimate of the relative transfection efficiencies. The transfection efficiencies were approximately equal. Cultures were then exposed to α-amanitin (10 μM; A), or a combination of α-amanitin and 5FU (375 μM; FA). At 48 h after treatment, the cultures were analyzed for cells with a sub-2n DNA content (shown in percent of cells) by flow cytometry. Error bars denote standard deviations from three experiments. Click here for file

Additional file 4 *Detection of p53-containing protein megacomplexes following BMH crosslinking in total cell extract but not mitochondrial fraction*. Detection of p53-containing protein megacomplexes following BMH crosslinking in total cell extract but not mitochondrial fraction. Total protein and mitochondrial cell extracts (15 μg) from HCT116 cultures expressing 273H were analyzed by standard SDS-PAGE after chemical crosslinking with BMH and subsequent immunoblotting of the complete gel (including wells and stacking gel) with anti-p53 antibody DO-1 at 1:2000 dilution. Total: total cell extract; mito: mitochondrial fraction. Click here for file
