# Supplementary figures and images for: Resistance of mitochondrial p53 to dominant inhibition
Source: Mol Cancer. 2008 Jun 12;7:54. doi: 10.1186/1476-4598-7-54 (PMC2440547; doi:10.1186/1476-4598-7-54)

**A**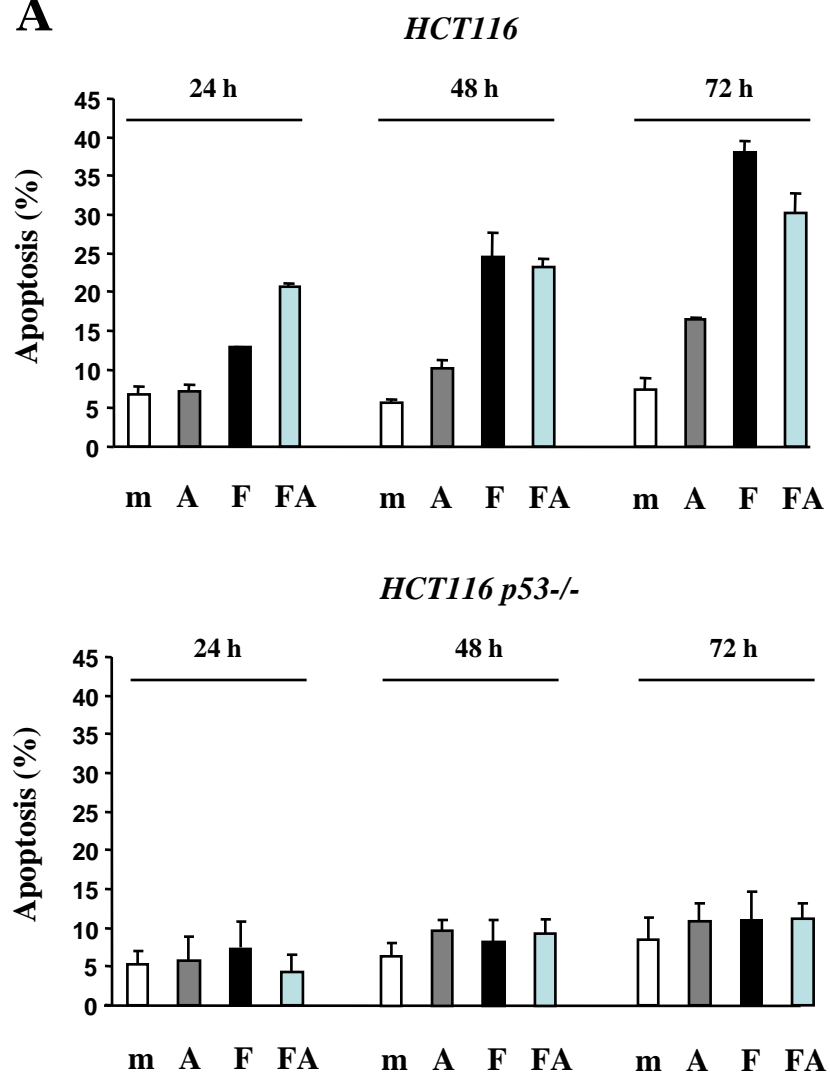**B**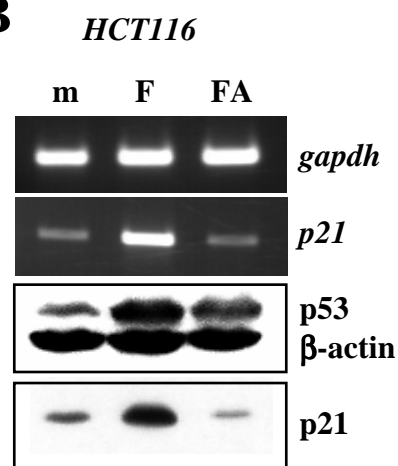**C**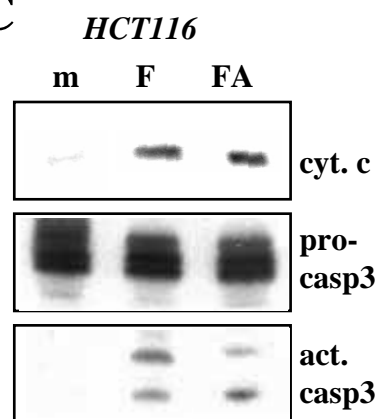

Supplement: Additional file 1 — α-amanitin and α-amanitin plus 5-fluorouracil can provoke a p53-mediated, transactivation-independent apoptosis in HCT116 cells. (A) Exponentially growing cultures of HCT116 and HCT116 p53-/- cells were mock-treated (m) or exposed to α-amanitin (10 μM; A), 5FU (375 μM; F), or both combined (FA). At the indicated time points, the percentage of apoptotic cells was determined by measuring the numbers of cells with a sub-2n DNA content after PI staining, as detailed in Materials and methods. (B) RT-PCR were performed on RNA from HCT116 cells after 24 h of drug treatment, as indicated, and the relative levels of the p53-responsive p21 and the control gapdh transcripts were determined. Western blot analysis confirmed the lack of stimulation of p21 under conditions of transactivation repression by α-amanitin. p53 was detected with DO-1 at a dilution of 1:2000; loading control β-actin was detected with anti-β-actin antibody diluted at 1:5000, and p21 was detected with anti-p21 antibody diluted at 1:1000. (C) Induction of apoptosis in HCT116 cells by 5FU and α-amanitin plus 5FU is mediated by cytochrome c (cyt.c) release and caspase 3 (casp3) activation. 15 μg of cytoplasmic protein (for cyt.c) or 30 μg of total protein (for casp3) from cells treated in the indicated way for 48 h were subjected to immunoblot analysis. Anti-cytochrome c antibody and anti-caspase 3 antibody (detecting both the pro-caspase and the activated caspase) were diluted at 1:1000. [file 1476-4598-7-54-S1.pdf]

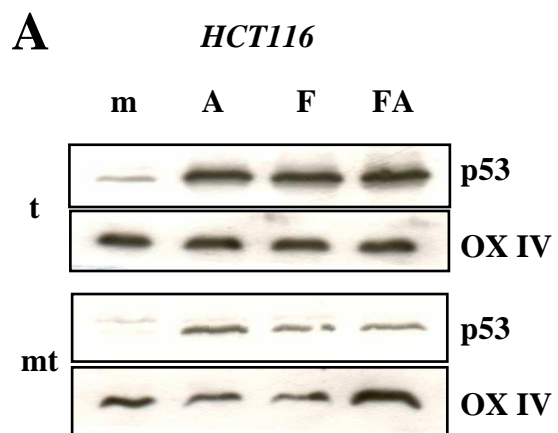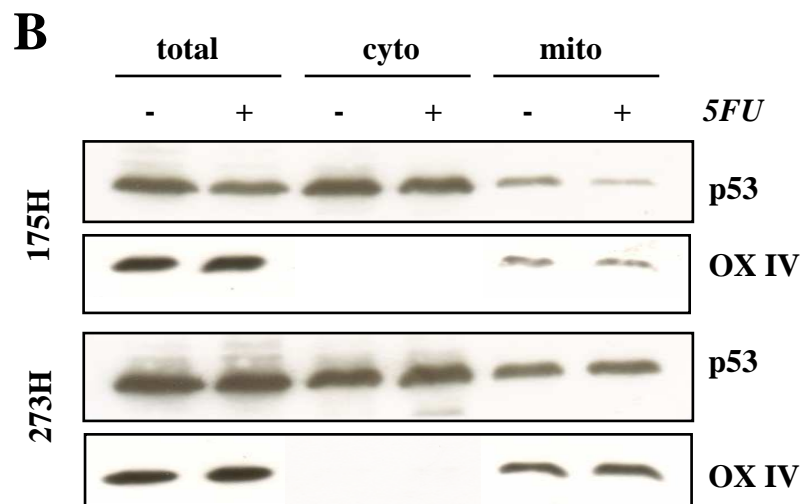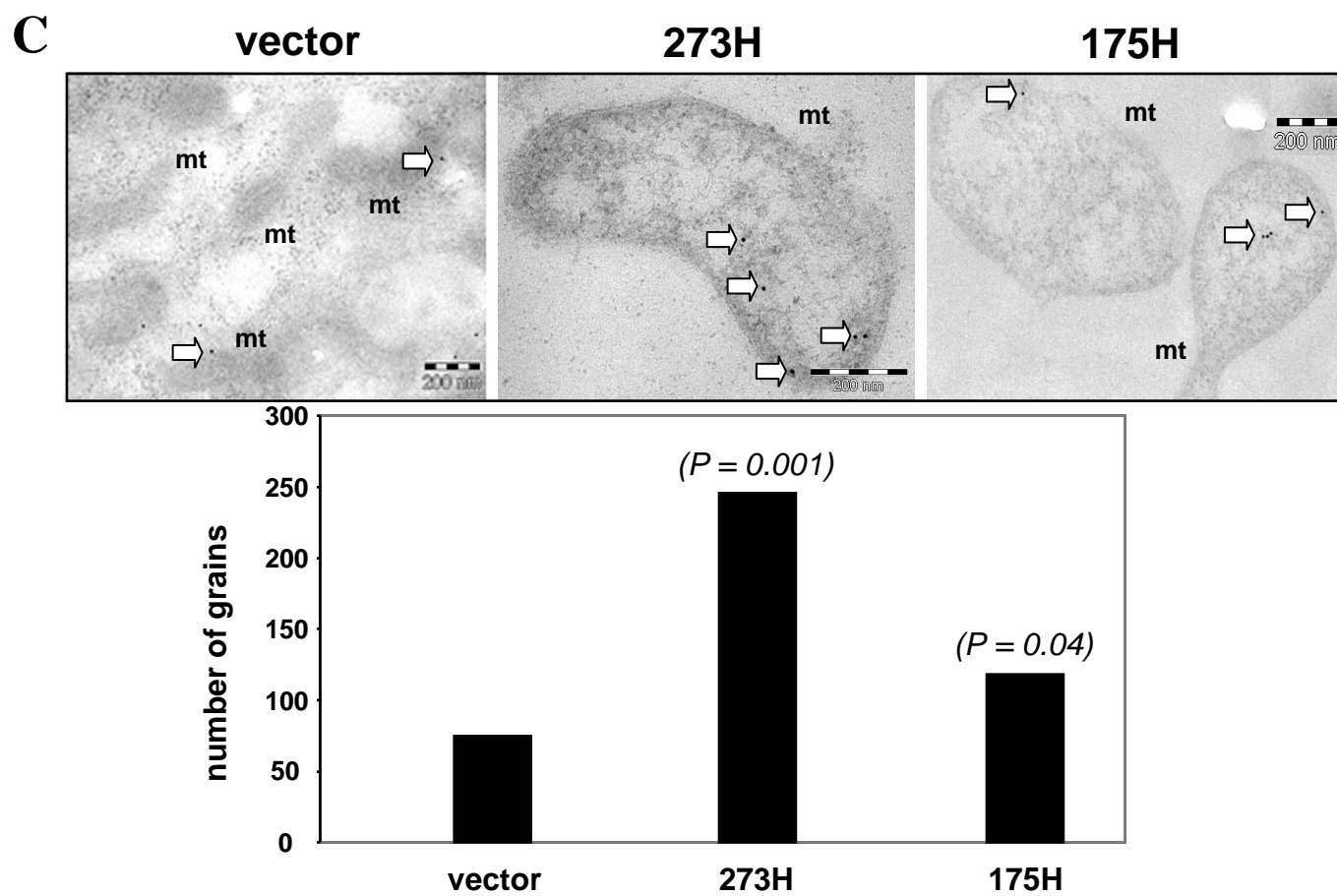

Supplement: Additional file 2 — Presence of wt and mutant p53 in the mitochondrial fractions of HCT116 cultures in dependence of treatment. (A) HCT116 cultures were mock-treated (m) or treated with 10 μM α-amanitin (A), 375 μM 5FU (F), or α-amanitin plus 5FU (FA) for 24 h; the cells were fractionated and the quality of the fractionation was tested as described in Materials and methods. 15 μg of total protein (t) or mitochondrial protein (mt) were subjected to Western immunoblot analysis with either anti-p53 antibody DO-1 (1:2000) or anti-cytochrome oxidase IV (OX IV) antibody (1:1000). (B) HCT116 p53-/- cells were bulk-infected with retroviruses at a multiplicity of infection of <1 pfu/cell to express either p53 full-length mutant 175H or 273H from single gene copies per cell. The cells were mock-treated (-) or treated with 375 μM 5FU for 24 h (+), and where then fractionated and analyzed by immunoblotting (15 μg protein). total = total cell extract; cyto = cytoplasmic; mito = mitochondrial fraction. Again, proteins were detected with anti-p53 and anti-cytochrome oxidase antibodies. (C) Immune electron microscopy detects mutant p53 at the mitochondria of HCT116 p53-/- cells retrovirally infected to express empty vector, 175H or 273H. Gold grains (10 nm; arrows) indicative of the binding of anti-p53 antibody DO-1 were more frequently detected at mitochondria (mt) in cells harboring mutant p53 than in cells with no p53. The diagram outlines the numbers of grains counted in a blinded study at 105 mitochondria from randomly chosen microscopic fields; P values were determined with the Chi-square test. [file 1476-4598-7-54-S2.pdf]

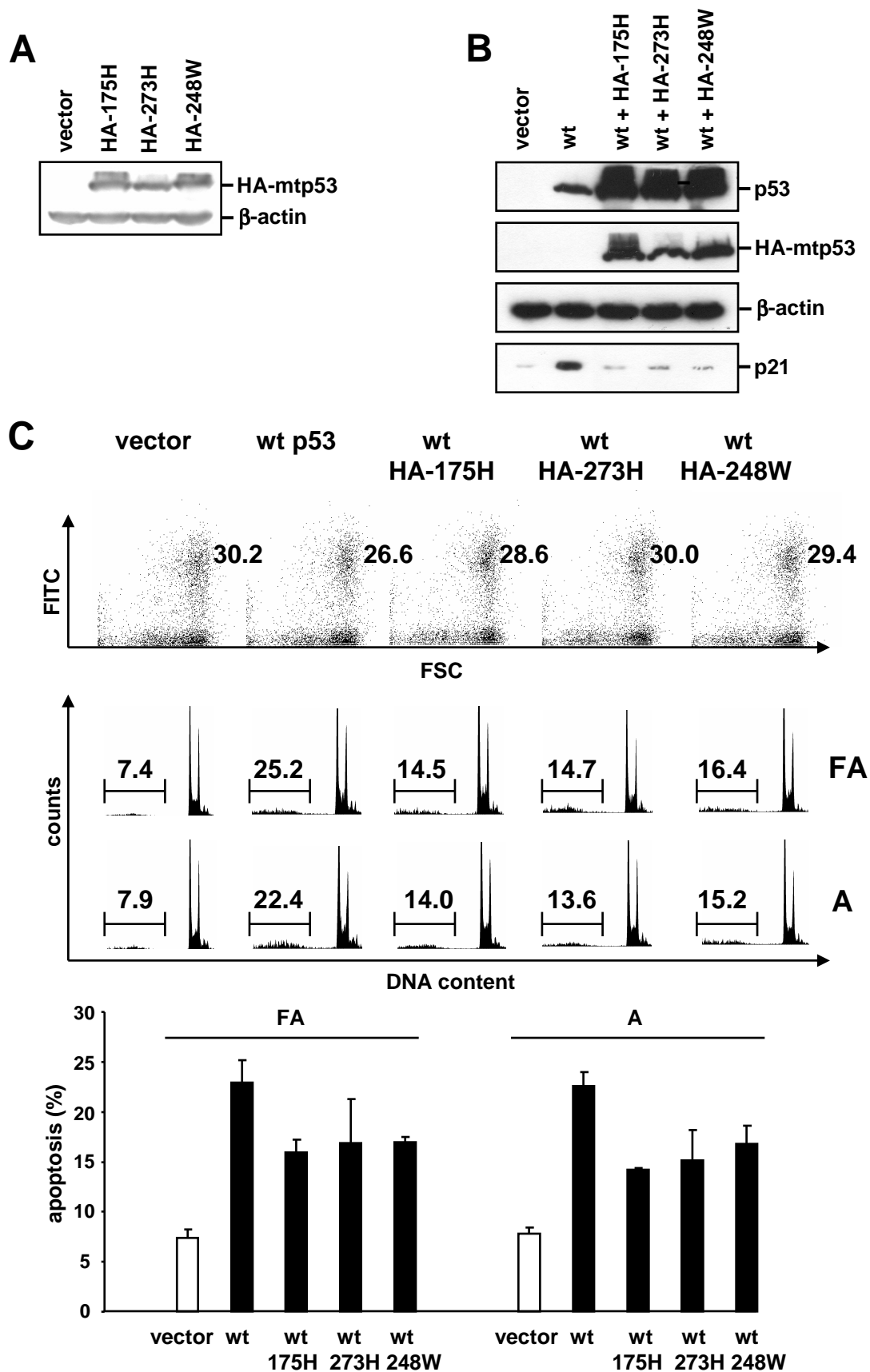

Supplement: Additional file 3 — Effect of full-length mutant p53 over-production on wt p53-mediated p21 expression and transcription-independent apoptosis. (A) HCT116 p53-/- cultures were transfected with empty vector or vector expressing HA-tagged full-length versions of 175H, 273H and 248W. p53 was detected with monoclonal anti-HA antibody 12CA5 (1:1000); loading control β-actin was detected with anti-β-actin antibody diluted at 1:5000. (B) Transient transfection of HCT116 p53-/- cultures with vector alone, or wt p53 plasmid plus vector plasmid or mutant p53 plasmid at a ratio of 1:3. p53 was detected with antibody DO-1 (1:2000); mutant p53 was detected with anti-HA antibody 12CA5 (1:2000). Loading control β-actin was detected with anti-β-actin antibody diluted at 1:5000. (C) Exponentially growing HCT116 p53-/- cultures were transfected as in B, but under inclusion of 0.1 μg plasmid expressing green fluorescent protein (pC-EGFP) to allow an estimate of the relative transfection efficiencies. The transfection efficiencies were approximately equal. Cultures were then exposed to α-amanitin (10 μM; A), or a combination of α-amanitin and 5FU (375 μM; FA). At 48 h after treatment, the cultures were analyzed for cells with a sub-2n DNA content (shown in percent of cells) by flow cytometry. Error bars denote standard deviations from three experiments. [file 1476-4598-7-54-S3.pdf]

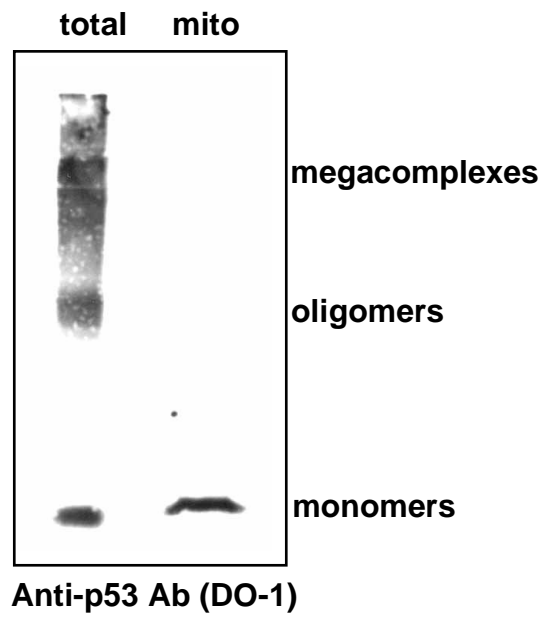

Supplement: Additional file 4 — Detection of p53-containing protein megacomplexes following BMH crosslinking in total cell extract but not mitochondrial fraction. Detection of p53-containing protein megacomplexes following BMH crosslinking in total cell extract but not mitochondrial fraction. Total protein and mitochondrial cell extracts (15 μg) from HCT116 cultures expressing 273H were analyzed by standard SDS-PAGE after chemical crosslinking with BMH and subsequent immunoblotting of the complete gel (including wells and stacking gel) with anti-p53 antibody DO-1 at 1:2000 dilution. Total: total cell extract; mito: mitochondrial fraction. [file 1476-4598-7-54-S4.pdf]
